# Oral *Candida* Carriage and Morphotype Differentiation in Chronic Periodontitis Patients with and without Diabetes in the Indian Sub-Continent

**DOI:** 10.3390/dj3040123

**Published:** 2015-11-02

**Authors:** Gomathinayagam Venkatesan, Ashita Uppoor, Dilip Naik, David Kadkampally, Abhiram Maddi

**Affiliations:** 1 Department of Periodontology, Manipal College of Dental Sciences, Manipal University, Mangalore, Karnataka 575001, India; E-Mails: dentistvenkatesan@gmail.com (G.V.); uash55@hotmail.com (A.U.); dilip.naik@manipal.edu (D.N.); david_mahe2005@yahoo.co.in (D.K.); 2 Department of Periodontics & Endodontics, School of Dental Medicine, State University of New York at Buffalo, Buffalo, NY 14214, USA

**Keywords:** periodontitis, diabetes, *Candida albicans*, yeast, hyphae

## Abstract

The aim of this study was to assess the oral *Candida* carriage and morphotype differentiation of *Candida* species in chronic periodontitis patients, with and without diabetes mellitus. This cross sectional study included 30 subjects in the age range of 40–60 years, who were divided into two groups: 15 chronic periodontitis only (CP) patients, and 15 chronic periodontitis patients with diabetes (CPD). Clinical measurements included plaque index (PI), gingival index (GI), probing depth (PD), clinical attachment level (CAL), and fasting blood sugar level (FBS). The unstimulated whole saliva samples were collected for fungal analysis. *Candida* carriage was analyzed by measuring colony forming units (CFU) following the culture of samples. Qualitative morphotype differentiation of *Candida* species from yeast to hyphal form was analyzed using Periodic acid-Schiff (PAS) staining. There was no statistically significant difference between CP and CPD groups for the periodontal parameters. However, a significantly higher *Candida* species CFU count was found in CPD (0.33 ± 0.23) as compared to CP (0.05 ± 0.04) group. This pilot study suggests that the occurrence of *Candida* species is higher in the saliva of chronic periodontitis patients with diabetes as compared to patients with chronic periodontitis alone.

## 1. Introduction

Periodontal disease is caused by the interaction between microorganisms, their products, and an exacerbated host immune response, resulting in the destruction of the tooth-supporting tissues. This process is associated with a widely diverse and complex subgingival microbiota, including both gram-positive and gram-negative bacteria that are either facultative or anaerobic organisms [1]. Despite the strong association of the so-called “Red Complex” bacteria with periodontitis, especially in the severe form of periodontitis, periodontal pockets can harbor a great variety of microorganisms, including yeasts [2,3].

*Candida albicans* is the most commonly occurring yeast in chronic periodontitis patients with a prevalence of 76.2% in the subgingival pockets [4]. *Candida* species are found as both yeast and hyphal morphologies. The yeast form of *Candida albicans* exists as planktonic cells that colonize epithelial surfaces. Change in environmental conditions influenced by local or systemic factors can trigger the yeast form to differentiate into hyphal form. Hyphae have the ability to penetrate and invade host tissues resulting in disease pathogenesis. Past studies have shown that *Candida* species are part of the subgingival microbiota of chronic periodontitis patients [2,4,5]. However, it is not clear if they contribute to the pathogenesis of periodontal disease.

The pathogenesis of periodontal disease requires the presence of bacterial plaque and a susceptible host. The requirement of a susceptible host for the development of periodontal diseases has led some to refer to periodontopathogenic bacteria as “required but not sufficient” to cause periodontal diseases. However, no disease process results from a single isolated cause or event, *i.e.*, no cause is necessary and sufficient in itself to produce disease [6].

*Candida* species are aerobic organisms that require sugar and a neutral to acidic pH for optimal growth. Oral anaerobic periodontal pathogens inhibit colonization and replication of *Candida* species, probably because anaerobes grow at and produce low oxygen tension, negative oxidation-reduction potential, and basic pH [7]. Despite this, *Candida albicans* is the mostly commonly found fungus in the subgingival pockets of chronic periodontitis patients [1,2]. This shows that *Candida albicans* has the capability to adjust to the anaerobic environment within the subgingival pocket.

*Candida* infections occur predominantly in immunocompromised patients with an underlying systemic condition. Hence, the presence of *Candida* species is significantly increased in patients with systemic disorders, such as diabetes mellitus, where the host susceptibility is increased [2]. In patients with diabetes, there is an increased level of serum glucose which results in several detrimental effects including increased risk for microbial infection and diminished host immune response.

It has been shown that *Candida albicans* carriage and density are increased in the oral cavities of diabetic patients [8]. There is also growing evidence that the number of yeasts is elevated in the periodontal pockets of diabetic patients [1]. Moreover, clinical studies have shown that there is an increase in salivary glucose levels in diabetes patients [9]. Diabetes and chronic periodontitis are chronic inflammatory diseases with a strong association. In this pilot study, we investigate whether increased glucose levels due to diabetes affect oral *Candida* carriage, and also the morphological differentiation to hyphae in chronic periodontitis.

## 2. Results

There were no significant differences between CP (chronic periodontitis) and CPD (chronic periodontitis with diabetes) patients with respect to the periodontal parameters including PI (plaque index), GI (gingival index), PD (probing depth) and CAL (clinical attachment loss) (Table 1).

**Table 1:** Comparison of periodontal parameters.

Parameters	CP	CPD	*p*-*Value* (*CP* vs. *CPD*)
PI	1.2 ± 0.34	1.09 ± 0.35	NS
GI	1.17 ± 0.43	0.92 ± 0.21	NS
PD	6.15 ± 0.84	6.29 ± 0.99	NS
CAL	6.36 ± 1.01	6.45 ± 1.18	NS

S—Statistical significance (*p* < 0.05); NS—Not significant; PI—Plaque Index; GI—Gingival Index; PD—Probing Depth; CAL—Clinical Attachment Loss; CP—Chronic Periodontitis; CPD—Chronic Periodontitis and Diabetes.

**Figure 1:**
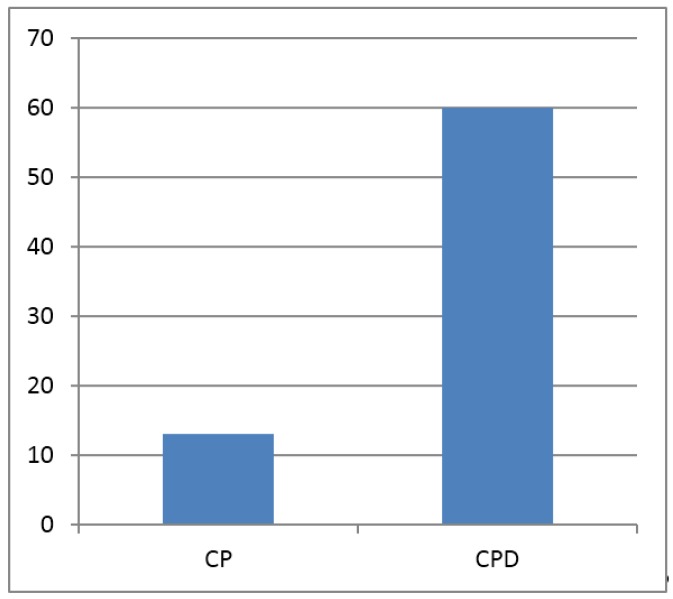
Mean occurrence of hyphal form of *Candida* in saliva of chronic periodontitis (CP) patients and patients with chronic periodontitis and diabetes (CPD).

The mean PD and CAL for the CPD group were higher than CP group but not statistically significant (Table 1). FBS (fasting blood sugar) values were significantly different between the groups, always with the highest values for the CPD group (Table 2). The number of *Candida* CFUs (colony forming units) was significantly higher for CPD group as compared to CP group (Table 2). A qualitative microscopic evaluation of the centrifuged saliva sample using PAS (Periodic acid-Schiff) staining techniques revealed the presence of multiple PAS positive filamentous structures that were suggestive of *Candida* hyphae. A total of 13% CP group patients showed the presence of hyphae form of the *Candida* species in their saliva whereas it increased to 60% for the CPD group patients (Figure 1).

**Table 2:** Fasting blood sugar and *Candida* colony forming units (CFU).

Parameters	CP	CPD	*p*-*Value* (*CP vs. CPD*)
FBS	97.33 ± 4.83	153.26 ± 47.37	S
CFU	0.05 ± 0.04	0.33 ± 0.23	S

S—Statistical significance (*p* < 0.05); NS—Not significant; FBS—Fasting Blood Sugar; CFU—Colony Forming Units; CP—Chronic Periodontitis; CPD—Chronic Periodontitis and Diabetes.

## 3. Discussion

The association between diabetes and inflammatory periodontal diseases has been studied extensively. There is evidence that patients with diabetes have a greater risk for periodontal disease [10]. Previous studies have shown that subjects with Type II diabetes have a three-fold increase in the risk for developing destructive periodontal disease as compared to healthy controls [11].

When comparing periodontal studies, differences may be detected in the clinical examination related to recording design, type, and number of sites assessed to measure PD and CAL. Although several partial mouth periodontal examination (PMPE) protocols produced small biases for estimates of disease severity and extent, they have shown various degrees of underestimation of disease prevalence [12]. Thus, in the present study, full mouth periodontal examination was carried out in six sites per tooth. In the present study, no significant differences in PI and GI were found when both genders were pooled together. There was no significant difference in PD and CAL between CP and CPD groups were observed concurrent with past studies [1,13].

*Candida albicans* is the most commonly found yeast in the oral cavity. It exists mainly in two morphologic forms: budding yeast (blastopore) form which is innocuous, and hyphal form [14]. The ability of *Candida* to adhere to the mucosa and dentures plays an important role in the pathogenesis of oral yeast infections. The adhesion of *C. albicans* to buccal epithelial cells (BEC) of diabetic patients was significantly greater than non-diabetics. This has been attributed to the increased expression of receptors for *Candida* species adhesion due to diabetic status [15].

Many studies have found that there is an increased incidence of *C. albicans* in the subgingival pockets of chronic periodontitis patients with diabetes [1,2]. *Candida* species possess virulence factors relevant in the pathogenesis of periodontal disease, such as the ability to adhere to the epithelium and invade the gingival connective tissue, the ability to inhibit the function of polymorphonuclear neutrophils (PMN), and the ability to produce enzymes such as collagenases and proteinases, which degrade immunoglobulins [5,16,17,18]. The microorganisms that are capable of degrading IgA (immunoglobulin A) may acquire a selective advantage in the colonization of oral surfaces. The proteolysis of immunoglobulins facilitates the penetration and spread of potentially toxic substances or antigens released by the subgingival microbiota. This could result in inflammatory changes associated with destructive periodontal diseases [19]. Such periodontal alterations have been considered to occur as a result of an exacerbated immune response against the host tissues, with changes in cellular and humoral immune responses. This may allow different microbial species, such as *C. albicans*, to colonize the subgingival environment [20].

However the exact mechanism of colonization of the subgingival pocket by *Candida* is not clearly understood. The transfer of *Candida* from the mucosal surface to the periodontal pockets may occur through saliva. The analysis of whole saliva is considered to be a sensitive approach for assessing oral *Candida* carriage [21]. Moreover, the transition of *Candida* from the yeast to hyphal forms is required for disease pathogenesis [2].

The detection of fungi in the saliva and subgingival region has been suggested to contribute to the pathogenesis of periodontal disease and to increase the possibility of candidiasis [7]. This may occur mainly due to systemic factors (e.g., the degree of glycemic control) or local factors (e.g., presence of dentures, xerostomia) that may influence the balance between the host and yeasts, and favor the transformation of *C. albicans* isolates from commensal to pathogenic microorganisms [22]. In the current study, the *Candida* CFU in whole saliva of CP and CPD group patients was analyzed. The results showed that CPD group had a higher prevalence of *Candida* CFU when compared to CP group (*p* < 0.02, Wilcoxon signed rank test). Additionally, in the present study PAS staining was done to demonstrate the presence of hyphal form of *Candida* species. Chronic periodontitis patients with diabetes had a greater prevalence of the hyphal form of *Candida,* explaining the increased conversion of yeast to hyphal form in the immunocompromised diabetic state. However, it should be noted that PAS staining may not differentiate *Candida albicans* with other species like *Candida tropicalis* and *Candida stellatoidea* which have morphological features similar to the former [21].

## 4. Materials and Methods

### 4.1. Study Population

The study protocol was approved by Institutional Ethical committee of Manipal College of Dental Sciences (MCODS), Mangalore, Karnataka, India. The patients for this study were recruited from the Department of Periodontics, MCODS who visited the out-patient department (OPD). All patients were informed of the protocol for the study and those who provided informed consent to participate in the study underwent periodontal examinations and provided whole saliva samples.

### 4.2. Inclusion and Exclusion Criteria

Patients were recruited according to the following inclusion criteria: subjects in the age group of 40–60 years, presence of at least 20 teeth, ≥5 mm of clinical attachment loss, probing depth >4 mm, diagnosed with Type I or Type II diabetes for at least 1 year with a history of oral hypoglycemic drug or insulin use. Exclusion criteria were as follows: a history of smoking, betel nut chewing, antibiotic therapy in past three months, systemic diseases other than diabetes mellitus, history of anemia, radiation therapy, and use of dentures.

### 4.3. Clinical Analysis

A total of 30 subjects were included in this study. The subjects were divided into two groups. Group 1 (CP): 15 subjects diagnosed with generalized chronic periodontitis without any underlying systemic conditions. Group 2 (CPD): 15 subjects diagnosed with generalized chronic periodontitis and diabetes. Of these, six subjects had Type I diabetes and were on insulin therapy and nine subjects had Type II diabetes. Periodontal destruction consistent with clinical attachment loss at >30% of sites were considered for a diagnosis of generalized chronic periodontitis [23]. The following periodontal parameters were measured: probing depth (PD), clinical attachment level (CAL), plaque index (PI), and gingival index (GI) by a calibrated single examiner, using a graduated William’s periodontal probe at six sites (mesio-buccal, mid-buccal, disto-buccal, disto-lingual, mid-lingual, mesio-lingual) per tooth. The periodontal parameters were determined for all teeth, excluding third molars. PD and CAL were calculated in units of millimeters.

### 4.4. Fasting Blood Glucose (FBS)

Subjects with diabetes reported the number of years since their diagnosis and insulin initiation as well as their daily consumption of insulin, and FBS was assessed for every patient. Subjects were asked to refrain from consumption of food or drink for 12 h before blood collection.

### 4.5. Collection of Whole Saliva

The subjects were asked to rinse their mouth with water before sample collection. The subjects were asked to sit in a dental chair with their head tilted forward and instructed not to speak, swallow or do any head movements during the procedure, or swallow any saliva present in the mouth. Then they were instructed to spit in a sterile graduated container every minute for 5 min. The whole saliva thus collected was transferred to a sterile container (stericol), capped, labelled and stored at −80 °C until biochemical analysis.

### 4.6. Microbial Culture and Analysis

The whole saliva samples were centrifuged for 10 min at 1000 rpm. The resulting supernatant was discarded and the pellet was re-suspended in 1 mL of sterile saline solution [24]. Following this, 20 µL of sample was taken and streaked onto Sabouraud dextrose agar plates and incubated at 37 °C for 48 h [21]. The number of colony forming units (CFU) per plate was counted and the total number of CFUs per mL of sample was calculated. A part of the saliva samples (100 µL) before centrifugation was stored in cytological fixative and subjected to Periodic acid-Schiff (PAS) staining. Up to 10 µL of the stained sample was placed on a slide and examined under a light microscope. The presence of dark staining germtubes and hyphae was used as a confirmation for *Candida albicans* [21]. This was done in the Department of Oral Pathology and Microbiology, Manipal College of Dental Sciences, Mangalore.

### 4.7. Statistical Analysis

The data was uploaded to MS Excel and statistical analysis was performed using SPSS Statistics 17.0. Age and periodontal parameters (PI, GI, PD, and CAL) were compared for CP and CPD patients using Student *t*-test and ANOVA respectively. Fasting glucose levels were compared with one way ANOVA test separately to evaluate the differences between the CP and CPD groups. For microbiological analysis, the number of *Candida* CFU was compared between the CP and CPD groups using the Chi-square test. Differences were considered statistically significant for *p* < 0.05.

## 5. Conclusions

This pilot study shows that the frequency of oral *Candida* carriage in chronic periodontitis patients is modifiable by a diabetic status. Additionally, the prevalence of hyphae, the virulent form of *C. albicans*, is greater in diabetics, although chronic periodontitis patients may still have the hyphal form of candida present in their saliva at lower levels. Thus, increase in the oral load of *Candida* species, as measured in whole saliva, may occur as a result of increased glucose levels due to the diabetic status. This, along with the increased transition to hyphae, may result in the increased colonization of *Candida* species in the periodontal pockets of diabetic patients. The presence of inflammatory cytokines and altered immune response in both chronic periodontitis and diabetes may influence this phenomenon. Further studies with larger patient populations are needed to understand the correlation between oral *Candida* carriage and subgingival *Candida* carriage to validate this hypothesis. Such studies might benefit from the use of species-specific biomarkers and genomic analyses to differentiate between the various species of *Candida*.

## Abbreviations

**CP**	chronic periodontitis	**CFU**	colony forming units
**CPD**	chronic periodontitis with diabetes	**PAS**	Periodic acid-Schiff
**PI**	plaque index	**OPD**	out-patient department
**GI**	gingival index	**PMPE**	partial mouth periodontal protocols
**PD**	probing depth	**BEC**	buccal epithelial cells
**CAL**	clinical attachment level	**PMN**	polymorphonuclear neutrophils
**FBS**	fasting blood sugar

## References

[1] Sardi J.C., Duque C., Camargo G.A., Hofling J.F., Gonçalves R.B. (2011). Periodontal conditions and prevalence of putative periodontopathogens and *Candida* spp. In insulin-dependent type 2 diabetic and non-diabetic patients with chronic periodontitis—A pilot study. Arch. Oral Biol..

[2] Canabarro A., Valle C., Farias M., Santos F., Lazera M., Wanke B. (2013). Association of subgingival colonization of *Candida albicans* and other yeasts with severity of chronic periodontitis. J. Periodontal Res..

[3] Maddi A., Scannapieco F.A. (2013). Oral biofilms, oral and periodontal infections, and systemic disease. Am. J. Dent..

[4] Cuesta A., Jewtuchowicz V., Brusca M., Nastri M., Rosa A. (2009). Prevalence of *Staphylococcus* spp and *Candida* spp in the oral cavity and periodontal pockets of periodontal disease patients. Acta Odontol. Latinoam..

[5] Järvensivu A., Hietanen J., Rautemaa R., Sorsa T., Richardson M. (2004). *Candida* yeasts in chronic periodontitis tissues and subgingival microbial biofilms *in vivo*. Oral Dis..

[6] Teles R., Teles F., Frias-Lopez J., Paster B., Haffajee A. (2013). Lessons learned and unlearned in periodontal microbiology. Periodontology.

[7] Hannula J., Dogan B., Slots J., Ökte E., Asikainen S. (2001). Subgingival strains of *Candida albicans* in relation to geographical origin and occurrence of periodontal pathogenic bacteria. Oral Microbiol. Immunol..

[8] Tapper-Jones L., Aldred M., Walker D., Hayes T. (1981). Candidal infections and populations of *Candida albicans* in mouths of diabetics. J. Clin. Pathol..

[9] Mascarenhas P., Fatela B., Barahona I. (2014). Effect of diabetes mellitus type 2 on salivary glucose—A systematic review and meta-analysis of observational studies. PLoS ONE.

[10] Mealey B.L. (2006). Periodontal disease and diabetes: A two-way street. J. Am. Dent. Assoc..

[11] Emrich L.J., Shlossman M., Genco R.J. (1991). Periodontal disease in non-insulin-dependent diabetes mellitus. J. Periodontol..

[12] Tran D.T., Gay I., Du X.L., Fu Y., Bebermeyer R.D., Neumann A.S., Streckfus C., Chan W., Walji M.F. (2013). Assessing periodontitis in populations: A systematic review of the validity of partial-mouth examination protocols. J. Clin. Periodontol..

[13] Yuan K., Chang C.J., Hsu P.C., Sun H.S., Tseng C.C., Wang J.R. (2001). Detection of putative periodontal pathogens in non-insulin-dependent diabetes mellitus and non-diabetes mellitus by polymerase chain reaction. J. Periodontal Res..

[14] Slutsky B., Buffo J., Soll D.R. (1985). High-frequency switching of colony morphology in *Candida albicans*. Science.

[15] Darwazeh A., Lamey P.J., Samaranayake L., MacFarlane T., Fisher B., Macrury S., MacCuish A. (1990). The relationship between colonisation, secretor status and *in vitro* adhesion of *Candida albicans* to buccal epithelial cells from diabetics. J. Med. Microbiol..

[16] Barros L.M., Boriollo M.F., Alves A.C.B., Klein M.I., Gonçalves R.B., Höfling J.F. (2008). Genetic diversity and exoenzyme activities of *Candida albicans* and *Candida dubliniensis* isolated from the oral cavity of brazilian periodontal patients. Arch. Oral Biol..

[17] Maccarinelli G., Belotti R., Savoldi E., Gervasoni M., Cocchi D. (2000). Phagocytosis and killing of candida albicans of polymorphonuclear cells in patients with organ transplant of periodontal disease. Miner. Stomatol..

[18] Reynaud A., Nygaard-Østby B., Bøygard G.K., Eribe E., Olsen I., Gjermo P. (2001). Yeasts in periodontal pockets. J. Clin. Periodontol..

[19] Hägewald S., Bernimoulin J.P., Köttgen E., Kage A. (2002). Salivary iga subclasses and bacteria-reactiveiga in patients with aggressive periodontitis. J. Periodontal Res..

[20] Zambon J.J., Reynolds H.S., Genco R.J. (1990). Studies of the subgingival microflora in patients with acquired immunodeficiency syndrome*. J. Periodontol..

[21] Williams D.W., Lewis M.A.O. (2000). Oral microbiology: Isolation and identification of *Candida* from the oral cavity. Oral Dis..

[22] Manfredi M., McCullough M., Al-Karaawi Z., Vescovi P., Porter S. (2006). *In vitro* evaluation of virulence attributes of *Candida* spp. Isolated from patients affected by diabetes mellitus. Oral Microbiol. Immunol..

[23] Armitage G.C. (1999). Development of a classification system for periodontal diseases and conditions. Ann. Periodontol..

[24] Samaranayake L., MacFarlane T., Samaranayake L.P., MacFarlane T.W. (1990). Host factors and oral candidosis. Oral Candidosis.

